# Using Integrative Analysis of DNA Methylation and Gene Expression Data in Multiple Tissue Types to Prioritize Candidate Genes for Drug Development in Obesity

**DOI:** 10.3389/fgene.2018.00663

**Published:** 2018-12-19

**Authors:** Qingjie Guo, Ruonan Zheng, Jiarui Huang, Meng He, Yuhan Wang, Zonghao Guo, Liankun Sun, Peng Chen

**Affiliations:** ^1^Department of Genetics, College of Basic Medical Sciences, Jilin University, Changchun, China; ^2^Key Laboratory of Pathobiology, Ministry of Education, Jilin University, Changchun, China; ^3^College of Clinical Medicine, Jilin University, Changchun, China; ^4^Department of Pathophysiology, College of Basic Medical Sciences, Jilin University, Changchun, China

**Keywords:** DNA methylation, obesity, association, gene expression, CpG

## Abstract

Obesity has become a major public health issue which is caused by a combination of genetic and environmental factors. Genome-wide DNA methylation studies have identified that DNA methylation at Cytosine-phosphate-Guanine (CpG) sites are associated with obesity. However, subsequent functional validation of the results from these studies has been challenging given the high number of reported associations. In this study, we applied an integrative analysis approach, aiming to prioritize the drug development candidate genes from many associated CpGs. Association data was collected from previous genome-wide DNA methylation studies and combined using a sample-size-weighted strategy. Gene expression data in adipose tissues and enriched pathways of the affiliated genes were overlapped, to shortlist the associated CpGs. The CpGs with the most overlapping evidence were indicated as the most appropriate CpGs for future studies. Our results revealed that 119 CpGs were associated with obesity (*p* ≤ 1.03 × 10^−7^). Of the affiliated genes, *SOCS3* was the only gene involved in all enriched pathways and was differentially expressed in both visceral adipose tissue (VAT) and subcutaneous adipose tissue (SAT). In conclusion, our integrative analysis is an effective approach in highlighting the DNA methylation with the highest drug development relevance. SOCS3 may serve as a target for drug development of obesity and its complications.

## Introduction

Since 1980, the incidence of obesity has increased throughout the world (Stevens et al., 2012; Ng et al., 2014). The onset of obesity involves the interaction between genetic and environmental factors (Contaldo and Pasanisi, 2004; Ussar et al., 2015). Genome Wide Association Studies (GWASs) have successfully identified many genetic variations associated with human complex diseases and provide crucial new insights about underlying molecular mechanisms (De La Vega et al., 2011; Fall and Ingelsson, 2014; Winham et al., 2014; Evangelou et al., 2018). Until now, the largest obesity GWAS study has identified 97 body mass index (BMI) associated loci (*P* ≤ 5 × 10^−8^) from up to 339,224 individuals. However, most of the genetic susceptibility remains unclear (Locke et al., 2015).

Existing evidence suggests that obesity is a result of interactions between genetic and environmental factors (Marti et al., 2008). DNA methylation provides a molecular mechanism for the interaction between the environment and obesity, in that it may affect individual susceptibility to obesity by altering the gene expression. In recent years, the association between DNA methylation and obesity has intensively been studied (van Dijk et al., 2015; Dhana et al., 2018; Wang et al., 2018). For example, a genome-wide DNA methylation association study in obesity that recruited 5,387 individuals, identified 278 CpGs associated with BMI (Wahl et al., 2017). The associated CpGs have provided wider insight in addition to previous genetic studies. On the other side, the numerous associated CpGs has made it difficult for functional investigations using cell and animal models.

In this study, we applied an integrative analysis approach, to prioritize genes with more relevance from several associated CpGs. Using this approach, we identified *SOCS3* as a promising candidate for mechanism studies and drug development. This approach can also be adapted to genome-wide DNA methylation studies of other diseases.

## Methods

**Figure 1:**
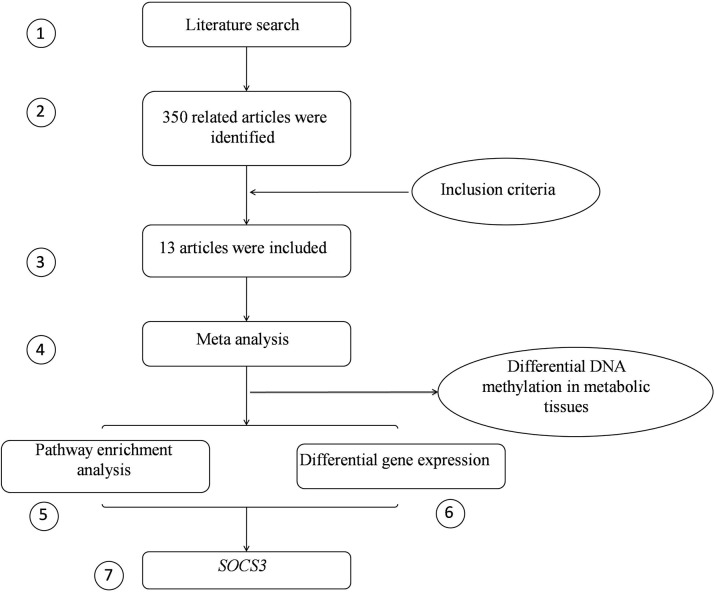
The flow chart of the intergrative analysis. The circled numbers represent the steps in the pipeline.

The integrative analysis approach included three components. The first component was to nominate the candidate CpGs by combining the association results from previous studies of peripheral blood samples (Steps 1–4, Figure 1). The second component was to estimate the functional relevance of the candidates through pathway enrichment analysis (Step 5). The third component was to validate that the genes affiliated with candidate CpGs were differentially expressed in adipose tissues (Step 6). Finally, the evidence from these components were put together and the genes with positive support from all components were considered and prioritized by our approach (Step 7).

### Literature Search

The literature search was conducted in the PubMed database using the keywords “CpG”, “DNA methylation” and “obesity” to capture all articles published from 2014 to 2018. We applied an English language restriction to our search results.

### Inclusion Criteria and Data Extraction

Both cohort studies and case-control studies reporting the association between DNA methylation and obesity (as measured by BMI) were included in this meta-analysis. Studies that used samples from cancer patients were not included. We further excluded the studies that used non-human subjects.

The full text of each article was carefully read to determine whether studies should be included. Once included, data were extracted from the articles, including the publication year, participant characteristics, sample size, association *p*-value, and the effect size.

### Meta-Analysis

We employed a sample-size weighted strategy to combine the *p* values reported in the included studies, taking into consideration the direction of the association effect size. This strategy was implemented using R software (https://www.r-project.org/). In this meta-analysis the CpG site with *p* value less than 1.03 × 10^−7^ (Bonferroni correction based on 485,577 CpGs designed in Illumina HM450K array) and with effect sizes consistent with the direction across all included studies, were considered as significant.

### Pathway Enrichment Analysis

We investigated the enrichment of the affiliated genes in the Kyoto Encyclopedia of Genes and Genomes (KEGG) pathways, using the Metascape online software (http://metascape.org; Tripathi et al., 2015). The genes were annotated using the default resources provided by Metascape. KEGG pathways were reduced using the default settings (the number of gene hits ≥3, enrichment *p*-value ≤ 0.05 and enrichment statistics ≥1.5). A FDR *p*-value ≤ 0.05 was taken to declare a significant enrichment.

### Differential Expression Analysis in Adipose Tissues

We aimed to investigate whether the associated genes were differentially expressed in the SAT and VAT of obesity patients, by comparing their gene transcription levels with normal individuals. This analysis was performed using the GEO2R tool (https://www.ncbi.nlm.nih.gov/geo/geo2r/) on two datasets, GSE2508 (10 obese vs. 10 lean) and GSE88837 (15 obese vs. 15 lean) for the SAT and VAT, respectively. The gene transcription levels were assayed using Affymetrix Human Genome U95 V2 and U133 arrays. The differential gene expression in obese samples was identified using the Bayesian estimation by GEO2R. Transcription level data of each sample was queried from the GEO database (Davis and Meltzer, 2007). Empirical Bayes statistics were calculated using the R package “limma” (Smyth, 2004; Ritchie et al., 2015). The fold change of DNA methylation was calculated using the group mean. *P* value ≤ 0.05 and |log_2_ (fold change)| ≥1 were used as criteria for differentially expressed genes. CpGs which were differentially expressed in both tissue types were identified as relevant loci.

### The DNA Methylation Associated With Obesity in Human VAT and Liver Tissue

The DNA methylation of the included studies was all measured in peripheral blood, but the DNA methylation in peripheral blood may be different from that in the metabolic tissues. To test whether the association in peripheral blood samples can be transferred into obesity related tissue, we tested the association of the significant CpGs in human VAT and liver tissue, using two GEO datasets, GSE88940 (10 obese vs. 10 normal VAT samples) and GSE65057 (8 obese vs. 7 normal liver samples), respectively.

## Results

### Characteristic of Individual Studies

According to the keywords “CpG”, “DNA methylation” and “obesity”, a total of 350 related articles were retrieved. Two hundred and seventy studies were excluded based on the title and abstract, as they were inconsistent with inclusion criteria, leaving 80 articles. Of those, 67 articles were excluded after a full-text review. As a result, 13 articles were included in the analysis. The reason for the exclusion of most articles was because they were functional studies in cells or animals. The basic characteristics of the included studies are detailed in Table 1.

**Table 1:** Characteristics of the included genome-wide DNA methylation studies.

**References**	**Corhort source**	**Ethnicity**	**Subjects**	**Body mass Index(kg/m^**2**^)^a^**
Dick et al., 2014	Cardiogenics consortium	European	459	25.9(3.6)
	MARTHA	European	339	24.2(4.4)
	KORA	European	1,789	28.1(4.8)
Pan et al., 2015	GUSTO cohort	Asian	991	1.3(0.1)^b^
Aslibekyan et al., 2015	GOLDN	EA	991	28(6)
	ARIC	AA	2,105	30(6)
Al Muftah et al., 2016	Qatari cohort	Caucasian	123	28.3(6.2)
	Twins UK cohort	Caucasian	810	27.8(5.2)
Main et al., 2016	EUGENE2 Consortium	Caucasian	137	27.9(6.0)
Wahl et al., 2017	LOLIPOP	Asian	2,680	27.6(4.4)
	EGCUT Asthma	European	173	22.8(3.0)
	EGCUT CTG	European	96	26.7(5.1)
	ALSPAC	European	701	26.6(5.3)
	Twins UK	European	338	26.7(5.0)
	RS-III	European	731	27.6(4.6)
	Life Lines Deep	European	752	25.4(4.2)
	Leiden Longevity	European	642	25.5(3.5)
	RS-BIOS	European	762	27.8(4.2)
	LOLIPOP	Asian	656	27.0(4.4)
Mendelson et al., 2017	FHS	European	2,377	28.3(5.4)
	LBC 1936	European	920	27.8(4.4)
	LBC 1921	European	446	26.2(4.0)
Koh et al., 2017	KoCAS	Asian	692	19.4(1.3)
Wang et al., 2018	EpiGO	AA	128	18.8(1.3)
	LACHY	AA	284	24.1(5.6)
	BP stress cohort	AA	228	31.4(8.6)
Dhana et al., 2018	RS	European	1,450	27.7(4.4)
Xu et al., 2018	Community volunteers	Mixed	510	24.5(2.9)

a *Values are shown as mean ± SD*.

b *BMI was derived as weight (g) divided by height^2^ (cm^2^)*.

*MARTHA, MARseille THrombosis Association; KORA, Cooperative Health Research; ARIC, Atherosclerosis Risk in Communities; GUSTO, Growing Up in Singapore toward Healthy Outcomes; FHS, Framingham Heart Study; EUGENE2, European Network on Functional Genomics of Type 2 Diabetes; ALSPAC, Avon Longitudinal Study of Parents and Children; RS-III, Rotterdam Study III; BIOS, Biobank-based Integrative Omics Studies; LOLIPOP, The London Life Sciences Prospective Population; LBC, Lothian Birth Cohort; KoCAS, Korean Child-Adolescent Cohort Study; EpiGO, Epigenetic Basis of Obesity-Induced Cardiovascular Disease and Type 2 Diabetes; LACHY: Lifestyle, Adiposity and Cardiovascular Health in Youth; BP, Blood pressure; RS, Rotterdam Study; AA, African American; EA, European American*.

### Meta-Analysis and Pathway Enrichment Analysis

A total of 13 articles were enrolled in our meta-analysis. The pooled peripheral blood samples for each CpG ranged from 700 to 18,370. We identified 119 CpGs associated with obesity, that reached a genome-wide significant level of *p* ≤ 1.03 × 10^−7^ (Supplementary Table 1). The top 10 associated CpGs are shown in Table 2.

**Table 2:** The top 10 associated CpGs in the meta-analysis.

**CpG**	***P***	***N***	**Dir**	**Gene^a^**
cg06500161	4.76 × 10^−122^	16737	+ + + + + + + + +	*ABCG1*
cg00574958	1.44 × 10^−98^	17748	— — — — — — —	*CPT1A*
cg11024682	5.01 × 10^−81^	17670	+ + + + + + + + + + +	*SREBF1*
cg07573872	2.22 × 10^−56^	18370	— — — — — — —	*SBNO2*
cg27243685	3.64 × 10^−55^	17274	+ + + + + + + + + +	*ABCG1*
cg18181703	1.73 × 10^−51^	13417	— — — — — — — — +	*SOCS3*
cg09349128	2.39 × 10^−51^	13694	— — — —
cg26403843	6.72 × 10^−46^	16737	+ + + + + + + + +	*RNF145*
cg04927537	2.64 × 10^−44^	16737	+ + + + + + + + +	*LGALS3BP*
cg06192883	2.51 × 10^−40^	16737	+ + + + + + + + +	*MYO5C*

a *The genes were annotated using the default resources provided by Metascape*.

*CpG, Cytosine-phosphate-Guanine; P, P-value; N, the total sample size of the corresponding CpG sites; Dir, direction of association with body mass index*.

Seventy-eight genes were annotated to be affiliated with these CpGs and used as the input to the pathway enrichment analysis. These associated genes were enriched in three KEGG pathways related to insulin resistance, adipocytokine signaling and TNF signaling. However, none of them were significant after multiple testing corrections (FDR *p* > 0.05). According to the KEGG, there is only one gene (*SOCS3*) which was involved in all three pathways (Table 3).

**Table 3:** Enriched KEGG pathway.

**Description**	***P***	**FDR**	**Gene symbols**
Insulin resistance	5.23 × 10^−4^	0.26	*CPT1A,RPS6KA2,SREBF1, SOCS3*
Adipocytokine signaling pathway	1.77 × 10^−3^	0.44	*CPT1A,SOCS3,ACSBG1*
TNF signaling pathway	6.27 × 10^−3^	1.00	*BCL3,MAP3K5,SOCS3*

*KEGG, Kyoto Encyclopedia of Genes and Genomes; P, P-value; FDR, false discovery rate*.

### Differential Expression of the Affiliated Genes

We analyzed 30 VAT samples and 20 SAT samples to assess the differential expression of the affiliated genes. A *p* ≤ 0.05 and |log_2_ (fold change)| ≥1 were used as criteria for differentially expressed genes. In the SAT, a total of 392 differentially expressed genes were obtained, of which 317 were up-regulated and 75 were down-regulated. On the other hand, there were 875 differentially expressed genes in the VAT, of which 406 were up-regulated and 469 were down-regulated. Among the genes affiliated with the 119 significant CpGs, *SOCS3* and *DOK2* were differentially expressed in the SAT of obesity patients, while seven genes (*SOCS3, PRR5L, ABCG1, BRDT, B3GNT7, ZNF710*, and *RARRES1*) were differentially expressed in the VAT of obesity patients. It is worth mentioning that the *SOCS3* gene was up-regulated in both human SAT (log_2_ fold change = 1.06, *p* = 9.23 × 10^−3^) and VAT (log_2_ fold change = 1.92, *p* = 8.52 × 10^−3^). The results are detailed in Supplementary Tables 2, 3.

### The Association With Obesity in Human VAT and Liver Tissue

Twenty VAT samples and 15 liver tissue samples were analyzed to test whether the association in the peripheral blood samples can be transferred into the metabolic tissues. The results revealed that seven CpGs in 119 associated CpGs were significantly associated with obesity (*p* < 0.05) in both the VAT and the liver tissue. The detailed results are shown in Table 4. Interestingly, most of them were associated with obesity in the opposite direction in the VAT and the liver tissue. For example, the CpG site cg07136133 was hyper-methylated in the VAT (log_2_fold change = 0.135, *p* = 0.014), but hypo-methylated (log_2_ fold change = −0.066, *p* = 0.013) in the liver of obesity patients.

**Table 4:** The association with obesity in human VAT and liver tissue.

**CpG**	**peripheral blood**			**VAT**		**Liver tissue**	
	***P***	**Dir**	**Gene^a^**	**Logfc**	***P***	**Logfc**	***P***
cg07136133	1.67 × 10^−35^	— — — — — —	*PRR5L*	0.135	0.014	−0.066	0.013
cg07037944	1.43 × 10^−19^	— — — —— —	*DAPK2*	0.059	0.026	−0.046	0.024
cg22891070	4.93 × 10^−18^	+ + + + + +	*HIF3A*	−0.140	0.008	−0.094	0.027
cg09554443	2.58 × 10^−17^	— — –	*CD247*	0.078	0.016	−0.038	0.035
cg00741986	1.38 × 10^−8^	— —	*TNIP2*	0.076	0.012	−0.065	0.016
cg15011409	2.23 × 10^−8^	+ + +	*ICAM5*	−0.073	0.009	0.067	0.013
cg03257930	6.24 × 10^−8^	— —		0.109	0.040	−0.056	0.010

a *The genes were annotated using the default resources provided by Metascape*.

*CpG, Cytosine-phosphate-Guanine; VAT, visceral adipose tissue; P, P-value; Dir, direction of association with body mass index; Logfc, log fold change*.

## Discussion

In this study, we identified 119 obesity-associated DNA methylations in human peripheral blood samples by combining results from previous studies. We further implemented the integrative approach highlighting *SOCS3* among the numerous associated genes as a promising drug target.

The role of *SOCS3* in obesity was strongly supported by our pathway enrichment analysis and the differential gene expression in the metabolic tissues. The pathway enrichment analysis is an efficient tool for drug target discovery (Aguirre-Plans et al., 2018). A gene which was involved in each or most of the enriched pathways may be situated in an essential position in the etiology of obesity. In our results, *SOCS3* was the only obesity-associated gene whose protein regulated all three of the most enriched KEGG pathways. SOCS3 suppresses the target proteins by promoting their ubiquitination and degradation. Those included insulin receptor substrates (IRS1 and IRS2) in the liver cells and the leptin receptor (LEPR) in adipocytes (Bjorbak et al., 2000; Eyckerman et al., 2000; Rui et al., 2002; Howard et al., 2004). In our study, *SOCS3* gene expression was up-regulated in both the VAT and SAT of obesity patients. This observation is in line with the increased insulin resistance found in morbid obesity patients and it further confirmed SOCS3 as a promising drug target (Mitrou et al., 2013; Dawson et al., 2014; Pucci et al., 2014).

Although involved in the insulin signaling pathway, the association between *SOCS3* DNA methylation and type 2 diabetes (T2D) has been under debate. In studies of a small sample size (*N* < 300), the *SOCS3* CpG was not associated with T2D (*p* > 0.05), with or without the adjustment of the BMI (Al Muftah et al., 2016; Dayeh et al., 2016). Furthermore, it is even associated with a BMI with the adjustment of T2D in one of the studies using the same cohort (Dayeh et al., 2016). In a study with 1074 incident T2D samples and 1590 controls, the *SOCS3* CpG was associated with incident T2D (*p* = 1.2 × 10^−7^) without the adjustment of the BMI (Chambers et al., 2015).

On the other hand, it has also been considered controversial whether the obesity-associated *SOCS3* CpG impacts the transcription level. It was demonstrated that the hypo-methylation at the associated *SOCS3* CpG may induce higher SOCS3 expression in peripheral blood mononuclear cells (Ali et al., 2016). One might think that this is probably transferable to other tissues, however, the hypo-methylation at this CpG was found to be related to lower gene expression in the human pancreatic islet but related to higher gene expression in adipose tissue (Dayeh et al., 2016). This apparently controversial evidence has indicated that the regulation of SOCS3 expression might be much more complex than we previously thought. Further investigation is necessary to uncover the tissue-specific modifier of expression regulation of this gene and to understand whether this helps to clarify the association between *SOCS3* DNA methylation and T2D.

DNA methylations in peripheral blood samples could be different from those in metabolic tissues, like adipose tissue and liver cells (De Bustos et al., 2009; Lovinsky-Desir et al., 2014). The conclusion derived from non-metabolic tissues should be validated in multiple metabolic tissues, before being used as evidence to support drug development or clinical trials. However, the GEO DNA methylation datasets in metabolic tissues had a small sample size. A statistical power analysis showed that we only had 9.5% power to detect a weak effect of DNA methylation on obesity using 20 samples. We hope that a better powered DNA methylation analysis in metabolic tissues could be taken into consideration in future integrative studies.

As compared to *SOCS3*, other genes showed a relatively weak relevance in our integrative analysis. The DNA methylation of the *ABCG1* gene was the top signal in peripheral blood samples. Unfortunately, the *ABCG1* gene was only differentially expressed in the VAT of obesity patients. The *CPT1A* gene was involved in two enriched pathways, but not differentially expressed in adipose tissues. Except for *SOCS3, CPT1A*, and *ABCG1*, other top 10 associated genes, shown in Table 2, lacked evidence of differential expression in adipose tissues and involvement in the enriched pathways. When taking a closer look at the 119 associated genes, we did have five additional differentially expressed genes in the VAT and one in the SAT. However, their priority was not supported by the enriched pathways.

The strength of this study lies in overlapping multiple lines of evidence to prioritize the candidate genes for drug target development, from the many associated DNA methylations. The integrated approach included genome-wide screening results in peripheral blood samples, pathway enrichment analysis, and differential gene expression in multiple adipose tissues. Screening obesity-associated CpGs in peripheral blood is remains the most practical way currently, as peripheral blood samples are abundant in many research groups. However, it should be noted that genomic DNA methylation can vary among different tissue types. For example, from our DNA methylation analyses in metabolic tissues, we observed the opposite direction of association at most of the associated CpGs (Table 4).

It should be noted that our study had limitations. Firstly, the association results from the included studies, came with various types of data transformation and statistical models, the effect sizes showed strong heterogeneity. We combined the *p*-values using a sample-size-weighted strategy, which is a flexible approach, but can also be inaccurate. The genome-wide screening of our pipeline could be improved when the individual DNA methylation data is available. Secondly, we analyzed the GEO datasets using the GEO2R tool. This tool was not able to adjust for the covariates, e.g., age and sex, which may be helpful in minimizing the effects from confounding factors. Finally, the included GEO datasets have much smaller sample sizes as compared to the genome-wide screening, which may have increased the false negative rate of our approach.

## Conclusion

In summary, we integrated multiple lines of evidence to reveal candidate genes for the treatment of obesity and its complications. Our study provided new insights on the interaction between obesity and the epigenome. Future studies are warranted to discover more potential drug targets using larger sample sizes from metabolic tissues, and to elucidate the mechanism of *SOCS3* DNA methylation interacting with obesity.

## Author Contributions

PC contributed conception and design of the study. QG, RZ, YW, ZG, JH, and MH were responsible for finding literature and extracting data. QG conducted data analysis and wrote the first draft of the manuscript. PC and LS contributed to manuscript revision and final approval for submission. All authors reviewed the manuscript and provided comments.

### Conflict of Interest Statement

The authors declare that the research was conducted in the absence of any commercial or financial relationships that could be construed as a potential conflict of interest.

## Abbreviations

ABCG1: ATP Binding Cassette Subfamily G Member 1
BMI: body mass index
CpG: Cytosine-phosphate-Guanine
FDR: false discovery rate
GWAS: Genome Wide Association Study
IRS1: insulin receptor substrate 1
IRS2: insulin receptor substrate 2
KEGG: Kyoto Encyclopedia of Genes and Genomes
LEPR: leptin receptor
SAT: subcutaneous adipose tissue
SOCS3: Suppressor Of Cytokine Signaling 3
T2D: type 2 diabetes
VAT: visceral adipose tissue.
