# Corrigendum: Using Integrative Analysis of DNA Methylation and Gene Expression Data in Multiple Tissue Types to Prioritize Candidate Genes for Drug Development in Obesity

**DOI:** 10.3389/fgene.2019.00571

**Published:** 2019-06-21

**Authors:** Qingjie Guo, Ruonan Zheng, Jiarui Huang, Meng He, Yuhan Wang, Zonghao Guo, Liankun Sun, Peng Chen

**Affiliations:** ^1^Department of Genetics, College of Basic Medical Sciences, Jilin University, Changchun, China; ^2^Key Laboratory of Pathobiology, Ministry of Education, Jilin University, Changchun, China; ^3^College of Clinical Medicine, Jilin University, Changchun, China; ^4^Department of Pathophysiology, College of Basic Medical Sciences, Jilin University, Changchun, China

**Keywords:** DNA methylation, obesity, association, gene expression, CpG

In the published article, there was an error regarding the affiliation for Liankun Sun. He should have the affiliation ^4^Department of Pathophysiology, College of Basic Medical Sciences, Jilin University, Changchun, China instead of the affiliation ^1^Department of Genetics, College of Basic Medical Sciences, Jilin University, Changchun, China.

The authors apologize for this error and state that this does not change the scientific conclusions of the article in any way. The original article has been updated.

